# Dietary supplements and beverages: Knowledge, attitudes, and practices among semi-professional soccer players in KwaZulu-Natal, South Africa

**DOI:** 10.17159/2078-516X/2022/v34i1a14018

**Published:** 2022-01-01

**Authors:** S Nyawose, R Naidoo, N Naumovski, AJ McKune

**Affiliations:** 1 Discipline of Biokinetics, Exercise, and Leisure Sciences. School of Health Sciences, University of KwaZulu-Natal, Durban, South Africa; 2 Faculty of Health, University of Canberra, Discipline of Nutrition and Dietetics, University of Canberra, Canberra, ACT, Australia; 3 Functional Foods and Nutrition Research (FFNR) Laboratory, University of Canberra, Ngunnawal Country, ACT, Australia; 4 Department of Nutrition and Dietetics, Harokopio University, Athens 17671, Greece; 5 Faculty of Health, Discipline of Sport and Exercise Science, University of Canberra, Canberra, ACT, Australia; 6 Research Institute for Sport and Exercise, University of Canberra, Canberra, ACT, Australia

**Keywords:** performance enhancement, sports drinks, anti-doping, ABC Motsepe League

## Abstract

**Background:**

The ingestion of dietary supplements and beverages is prevalent in soccer, at the amateur and professional level. The absence of professional advice at non-professional level makes amateur soccer players susceptible to ingesting unsafe supplements.

**Objectives:**

To determine the knowledge, attitudes and practices of ABC Motsepe League (semi-professional) players in KwaZulu-Natal regarding the use of dietary supplements and beverages.

**Methods:**

Three hundred and forty-three soccer players participated in a cross-sectional study. Knowledge, attitudes and practices were determined using a questionnaire. Researchers visited twelve teams. On the day of the visit to each team, information sheets and questionnaires were given to participants. Questionnaires were collected immediately following completion. Descriptive statistics were used, including means and standard deviations, where applicable. Inferential statistics, Chi-square and binomial tests were used to analyse the results. Statistical significance was set at p < 0.05.

**Results:**

Sports beverages were the most recommended and commonly used, followed by energy beverages. Dietary supplements were the least-known used. Participants used beverages and dietary supplements to assist in providing more energy (67%), improve health (65%) and improve performance (55%) (p<0.001). Seventy-three percent of participants lacked knowledge about the anti-doping policy (p<0.001), with 87% having never attended a workshop on the safe use of supplements and beverages, or anti-doping awareness campaigns (p<0.001). Thirty-eight percent had not heard of the South African Institute for Drug-Free Sport (SAIDS), and 84% were not familiar with the yearly updated World Anti-Doping Agency’s (WADA) prohibited list (p<0.001). Of the 59% who did not take dietary supplements or beverages, 75% had insufficient information regarding them (p<0.001), 66% indicated that dietary supplements and beverages were costly (p=0.001), and 55% indicated they did not need dietary supplements and beverages (p=0.32).

**Conclusion:**

There is a need for an educational programme on the safe use of dietary supplements, and sports and energy beverages among KwaZulu-Natal semi-professional soccer players.

Dietary supplements include food components, nutrients and non-food compounds ingested in addition to a habitually consumed diet to achieve specific health and/or performance benefits.^[1]^ For the purpose of this study, these dietary supplements are typically in the form of tablets, capsules, soft gels, energy bars, and powders. Soccer players commonly consume dietary supplements, and sports and energy beverages, before, during, or after a soccer match.^[2]^ Sports beverages, are typically ingested to hydrate and restore electrolytes and carbohydrates; whereas energy beverages have caffeine as the main ingredient, with the goal of improving cognitive ability.^[3]^

Soccer is a competitive sport that involves intermittent, high-intensity activities interposed with low-intensity activities.^[3]^ A decline in any performance component can decide the outcome of a soccer match. Therefore, the strategic use of substances that improve hydration, energy and cognitive ability to enhance performance is attractive to soccer players.^[4]^

Commercially available dietary supplements and beverages appeal to athletes for their supposed ergogenic effect, mainly due to aggressive marketing strategies.^[5]^ Studies have reported widespread usage of dietary supplements in soccer players. More than 82% of dietary supplement usage has been reported among elite soccer players recruited from international tournaments.^[6]^ At the summit of world soccer, a retrospective study reported an increase in dietary supplement use during Russia's 2018 World Cup tournament, compared to the 2014, 2010, 2006, and 2002 World Cup tournaments.^[7]^

Beverages are consumed at any time during a soccer match, with consumption of energy beverages by soccer players primarily before or during a game.^[2]^ Sports and energy beverages are also commonly known about and used by amateur athletes.^[8]^ This increasing trend in the use of supplements is also evident in African countries. Fifty-one per cent of soccer players in the Zimbabwean league used dietary supplements, mainly vitamins (20%) and minerals (17%); while 15% reportedly consulted traditional healers for herbal supplements.^[9]^ All 200 participants in an Algerian study indicated that they used dietary supplements to improve performance.^[10]^ South African athletes, including amateur soccer players, are no different, with the vitamin and supplement market in South Africa growing at around 11% annually.^[11]^ Their use is more evident in elite athletes than in non-professional athletes. However, limited research has been conducted to compare use at the different performance levels.

Non-professional athletes typically have limited professional support to guide them in the safe use of supplements.^[12]^ The prevalence of supplement usage among non-professional athletes potentially increases the risks of using contaminated supplements. The frequent use of supplements is associated with a high risk of ingesting contaminated substances that may be harmful.^[1]^ Fifteen to 25% of supplements contain substances prohibited by World Anti-Doping Agency (WADA).^[2]^ Furthermore, many supplements come with insufficient scientific research or supporting evidence.^[13]^

Over the years, there have been cases against soccer players using prohibited substances in the South African premier division. In the province of KwaZulu-Natal, amateur soccer players use supplements, yet lacked knowledge about the risks associated with using these supplements.^[8]^ This may lead to non-professional soccer players in KwaZulu-Natal becoming susceptible to using prohibited substances.

To the best of the authors’ knowledge, there is currently no published data on the knowledge about, attitudes to, and practices of, dietary supplement and beverage use among semi-professional soccer players in South Africa. Therefore, this study aimed to determine the knowledge and use of dietary supplements and beverages among ABC Motsepe league players in KwaZulu-Natal.

## Methods

### Participants and study design

This study used a descriptive, observational, cross-sectional survey design. The researchers relied on a self-administered questionnaire to explore the participants’ practices and perceptions.

The ABC Motsepe League usually features 144 teams, divided into nine 16-team provincial divisions. Twenty teams were affiliated with the KwaZulu-Natal League during the 2021–2022 season. Teams affiliated to the KwaZulu-Natal League were approached to participate in the study. Sixteen teams were contacted telephonically, with thirteen accepting the invitation to participate. However, one team was not available on the day the researchers visited the teams for data collection. Therefore, 12 teams participated in the study. Three hundred and forty-three participants completed questionnaires. This meant a 95% questionnaire response rate.

Using a margin of error of 5% and an alpha level of .05 from a population of 600 the required sample size would be 234. Cochran’s sample size formula for categorical data was used to calculate the study’s sample size, and for a population size of 600, Cochran’s correction formula was used to calculate the final sample size.^[14]^

### Data collection

The dietary supplement questionnaire was used to determine knowledge, attitudes and practices regarding dietary supplements and beverages among ABC Motsepe League players in KwaZulu-Natal. The questionnaire was adapted from a reliable and validated questionnaire.^[12]^ The questionnaire was explained to participants in English and isiZulu. The questionnaire was comprised of four sections. Section A focused on general information; Section B focused on knowledge about dietary supplements and sports and energy beverages; while Section C focused on attitudes to dietary supplements and sports and energy beverages. Section D focused on the use of dietary supplements and sports and energy beverages. This section was divided into two subsections: one for participants who used supplements and the other for participants who did not. A pilot study was conducted with 12 players who played one division lower than the study population. Based on positive feedback from the participants, no changes were necessary.

### Ethical considerations

The University of KwaZulu-Natal’s Biomedical Research Ethics Committee (00001656/2020) approved the study. The South African Football Association’s (SAFA) KwaZulu-Natal Provisional Executive Council granted permission to conduct the research and provided a list of teams with the contact details of the team managers. Researchers visited twelve teams. On the day of the visit to each team, information sheets were handed out to prospective participants. The purpose of the study was explained in detail to the soccer players in the presence of the team management and technical team. Players interested in participating in the study signed consent, or assent forms for those under 18 years were completed, and the team manager signed consent. Questionnaires were handed out to participants, completed, and collated on the same day.

### Statistical analysis

Data were analysed using the statistical package for social science (SPSS) 21.0 (IBM Corp. Released 2013. IBM SPSS Statistics for Windows, Version 22.0. Armonk, NY: IBM Corp). Descriptive statistics were used, including means and standard deviations, where applicable. Inferential statistics, including the chi-square goodness of fit test, were used to perform univariate analysis on categorical variables to test whether any of the response options were selected significantly more or less often than the others. Sample size varied as a consequence of participants not completing all questions. Statistical significance was set at p<0.05.

## Results

Participants’ ages ranged from 16 to 35 years. The mean age was 24 ± 4 years, with 60% having played in the ABC Motsepe League for less than four years and 40% playing for more than four years. Of the players, 34% played in the midfield positions; 31% played as defenders; 24% played forward positions; and 11% were goalkeepers.

### Knowledge about dietary supplements and beverages

When participants were asked to indicate agreement with the statement that consuming supplements replaces a healthy balanced diet, results showed neither significant agreement nor significant disagreement (p=0.28).

Table 1 lists the supplements and beverages that ABC Motsepe League soccer players in KwaZulu-Natal consider ideal for soccer players, based on their knowledge. A significant number of the participants responded that soccer players' sports beverages were ideal for consumption: 74% recommended carbohydrate sports beverage A and 61% recommended carbohydrate sport beverage B (p<0.001). A significant number of participants did not recommend supplements, such as creatine (84%), nitrate (86%), or sodium bicarbonate (92%), as ideal for consumption by soccer players (p<0.001). However, 51% of players recommended protein shakes as an ideal supplement for soccer players (p=0.75).

### Knowledge of anti-doping agencies

Table 2 presents participant responses relating to their knowledge of anti-doping agencies: the World Anti-Doping Agency (WADA) and the South African Institute for Drug-Free Sport (SAIDS). A significant number of participants (73%) had no access to anti-doping information, while 87% had never attended a workshop or presentation on dietary supplement or beverages, or an anti-doping awareness campaign (p<0.001). A significant number of participants (84%) were not familiar with the yearly updated WADA prohibited list (p<0.001). A significant proportion of participants, 38% and 48%, had never heard of the SAIDS or WADA, respectively. While 35% and 30% had heard of SAIDS and WADA, respectively, they did not know anything substantial (p<0.001).

### Attitudes to dietary supplements and beverages

When questioned on their attitudes to dietary supplements and beverages, 73% disagreed that all supplements are safe, and 59% disagreed that taking dietary supplements is cheating (p<0.001). Participants (70%) agreed that it was necessary to read the nutritional guidelines for a dietary supplement or beverage product (p<0.001), while 67% agreed that the type, quantity, and timing when taking dietary supplements or beverages was essential to receive the full benefits from that product (p<0.001). There were no significant differences in participants' responses when asked if the claimed benefits of dietary supplements and beverages are always based on scientific evidence (p=0.05).

### Practices of dietary supplement and beverage use

When asked about the beverages or supplements that participants had consumed the most in the previous month, sports beverages were the most consumed (23%), followed by energy beverages (6%), dietary supplements at 5%, and protein shakes were used by 2.8%. However, 0.7% of participants indicated they did not know the beverage name or dietary supplement they had consumed most in the previous month. There was no significant difference between players who consumed beverages before training or matches (57%) and players who did not consume beverages (43%), (p=0.92). Of the participants, a significant 34% indicated that they followed the instructions on the beverage label or supplement label to help them decide on the amount to consume, while a significant 50% were unsure of how much to drink (p<0.001). A significant number of participants did not consult a professional (71%), ask a coach or teammates (72%), check the manufacturer’s websites (82%), or conduct any research (88%) about the safety of beverages or dietary supplements consumed (p<0.001).

### Reasons for using dietary supplements and beverages

Study participants were asked to recall if they had consumed dietary supplements or beverages in the month prior to data collection. Of the participants, 59% reportedly had not consumed dietary supplements or sports and energy beverages in the previous month, while 41% (n = 141) indicated that they had consumed dietary supplements and beverages in the month before data were collected.

**Fig. 1:**
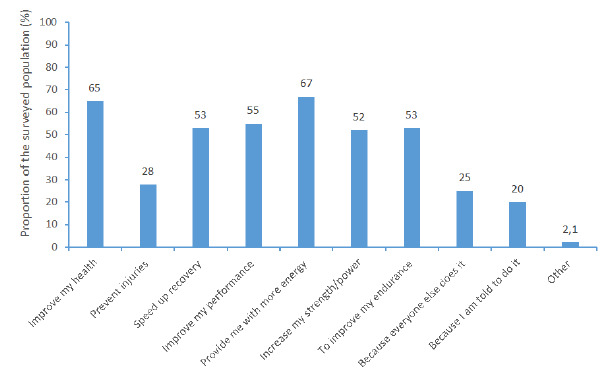
Reasons for using dietary supplements and beverages (%) reported by participants (n=141)

Figure 1 lists reasons for consuming dietary supplements, and sports and energy beverages. Participants used dietary supplements or beverages mainly to provide more energy (67%), improve health (65%) (p<0.001), and enhance sports performance (55%) (p=0.31). Study participants indicated that they mainly relied on online sources (38%) and coaches (36%) for information on dietary supplements and beverages.

Table 3 lists participants’ reasons for not taking supplements or sports and energy beverages. Participants disagreed that the consumption of supplements and beverages is a form of cheating (p < 0.02) and disagreed that supplements and beverages are unhealthy (p < 0.003). Participants agreed that supplements and beverages are often too expensive and that they are concerned about failing a drug test if they were to be tested (p < 0.05). Furthermore, participants agreed that they do not know enough about supplements and beverages (p <0.001).

## Discussion

The study aimed to determine the knowledge, attitudes, and practices of the ABC Motsepe League (semi-professional) soccer players in KwaZulu-Natal regarding the use of dietary supplements and beverages. There are limited studies that have focused on elite soccer players.^[15]^ Similarly, there is a lack of published literature on dietary supplements and beverages in the South African professional, semi-professional and non-professional leagues. Some of the information discussed below has been gathered from different levels of sport and recreation.

Dietary supplements are not substitutes for a balanced nutritional programme for athletes.^[1]^ However, responses from the current study showed neither significant agreement nor significant disagreement with the statement that ‘taking dietary supplements replaces a healthy balanced diet’. Furthermore, of the participants who had not recently used supplements or beverages, the majority reportedly did not know enough about beverages and supplements. This suggests that the participants had relatively limited knowledge about dietary supplements. Similarly, a study by Jovanov et al. reported that less than 40% of amateur athletes knew about the proper intended use of dietary supplements.^[12]^ However, professional players are relatively better informed about dietary supplements than amateur players.^[16]^ A study on soccer players in the professional Malawian Super League reported an adequate understanding of supplements, with no need for educational interventions among the players.^[17]^ This may indicate that at the professional level adequate education on dietary supplements is known, compared to lower divisions. Differences in resources, such as the availability of professional advice and finances, may account for the reportedly superior knowledge in the professional game, compared to non-professional players.

Among athletes from the recreational to elite levels, commercially available sports beverages are popular because of their marketed ergogenic effect.^[5]^ Similarly, in the current study, sports beverages were the most recommended as ideal for improving soccer players' performance. Protein shakes were the second most known and recommended supplement to enhance the performance of soccer players, preferred to other supplements, such as creatine, nitrate and sodium bicarbonate. A similar interest in, and popularity of, protein supplements was reported in Johannesburg gym attendees.^[18]^

Maughan et al. cautioned athletes to assess whether minor benefits from dietary supplements exceed the risks of unintentional doping.^[1]^ The responses of the semi-professional league soccer players in the present study showed a substantial lack of knowledge of the WADA list of prohibited substances, which suggests that players are at a high risk of using prohibited substances. A similar knowledge gap was reported in a recent scoping review on non-professional players, especially with regard to the health risks associated with consuming supplements.^[19]^ Furthermore, most players had limited to no knowledge about anti-doping agencies, such as SAIDS and WADA. Lack of proper guidance on the risks associated with supplements increases the risk of doping and its adverse effects on the athletes’ health.^[2]^

Despite the players indicating that they are not fully knowledgeable about the risks associated with consuming dietary supplements, their attitude to dietary supplements seemed to indicate otherwise. Participants disagreed that all supplements are safe for consumption, and the majority of participants (59%) indicated that they did not consume supplements because they considered them unhealthy. This attitude is supported by a study that advised exercising caution when using supplements or beverages as any compound that may enhance performance has the potential to adversely affect one’s health.^[1]^ However, in the current study it may be possible that participants were not familiar with the names of supplements or beverages.

Despite the cautious attitudes regarding the consumption of dietary supplements and sports and energy beverages displayed by some participants, it is well known that supplementation is common among soccer players.^[1]^ Forty-one per cent of participants used sports beverages to enhance their performance during soccer matches or training.^[1]^ Similarly, a study by Coopoo et al. reported that large quantities of carbohydrate supplements were consumed by gym attendees.^[18]^ Sports beverages promote hydration by replenishing electrolytes and supplying carbohydrates.^[20]^ The strategic use of substances that improve hydration is deemed important in soccer players, given the few opportunities to hydrate during a soccer match.^[2]^

Energy beverages were the second most consumed beverage in this study. This may be due to the benefits attributed to caffeine-containing beverages in improving gross motor skills and concentration during a match.^[21]^ A study on amateur soccer players reported that coaches recommended energy drinks to players.^[8]^ In the current study, participants mainly relied on online sources and coaches for advice in choosing a supplement for use. Athletes frequently use caffeine-containing beverages before a soccer match to enhance performance.^[2]^ The present study found that 57% of participants consumed beverages before a match or training session. However, it may be of concern that soccer players believed that drinking more is better in order to have an edge over the opposition. Non-professional athletes might be susceptible to excessive energy beverage consumption by thinking that this will increase their performance further.

### Limitations

The study’s findings should be interpreted cautiously, since the study used a self-administered questionnaire. It is essential to acknowledge the possibility of recall bias while responding to the questionnaire, and the likelihood of not providing an honest answer. Players’ understanding of terminology in the questionnaire may have been another limitation. However, the questionnaire was explained to all participants in English and IsiZulu.

## Conclusion

The KwaZulu-Natal ABC Motsepe soccer players displayed a cautious attitude to using dietary supplements and sports and energy beverages possibly because of the evidence, and substantial lack of knowledge about the proper consumption of supplements. Furthermore, users of supplements in this study were at risk of unintentional doping because of their limited or no access to educational platforms from anti-doping agencies, such as SAIDS and WADA. There is an urgent need for educational programmes to promote awareness about the safe use of dietary supplements and sports and energy beverages in the KwaZulu-Natal ABC Motsepe League. Such programmes should teach soccer players about prohibited substances, thus avoiding unintentional doping and the potential side effects that impact health.

## Figures and Tables

**Table 1:** Supplements, and sports and energy beverages, ideal for soccer players (%), as reported by participants (n=343)

Item	Frequency (%)		p-value

	Yes	No	
Caffeine	22	78	<0.001**
Creatine	16	84	<0.001**
Red Bull®	41	59	0.001*
Nitrate	14	86	<0.001**
Protein shakes	51	49	0.75
Carbohydrate beverage A (Powerade^®^)	74	26	<0.001**
Carbohydrate beverage B (Energade®)	61	39	<0.001**
Sodium bicarbonate	8	92	<0.001**

* indicates p < 0.05,

** indicates p< 0.001.

**Table 2:** Knowledge of anti-doping agencies (%) reported by participants (n=343)

Item	Responses as Frequency (%)					Χ^2^	df	p-value

	Never heard of it	Heard of, know nothing about it	Heard of, know a little about it	Heard of, know quite a bit about it	Heard of, know a lot about it			
SAIDS	38	35	20	3.8	3.5	186.8	4	<.001*
WADA	48	30	13	7.6	2.0	239.5	4	<.001*

* indicates p< 0.001.

SAIDS, South African Institute for Drug-Free Sport; WADA, World Anti-Doping Agency; X^2^, chi-square test; df, degrees of freedom

**Table 3:** Reasons for not using dietary supplements or sports and energy beverage

Reasons	n	Score	t	df	p-value
I do not need supplements and beverages.	196	3.6 ± 1.7	0.99	195	0.32
They are unhealthy.	196	3.2 ± 1.5	−2.98	195	0.003*
They are too expensive.	196	3.9 ± 1.5	3.48	195	0.001*
I do not know enough about supplements and beverages.	195	4.3 ± 1.4	7.93	194	<0.001**
I am concerned about a positive drug test.	196	3.8 ± 1.6	2.98	195	0.003*
Taking supplements or energy beverages is like cheating.	196	3.2 ± 1.6	−2.35	195	0.020*

* indicates p < 0.05,

** indicates p< 0.001.

Scored on Likert scale from 1 to 6. Score expressed as mean ± standard deviation. Results were interpreted as significant agreement if the mean score >3.5 or significant disagreement if the mean score <3.5. df, degrees of freedom.
